# An epidemiological investigation of age-related macular degeneration in aged population in China: the Hainan study

**DOI:** 10.1007/s10792-017-0639-7

**Published:** 2017-07-07

**Authors:** Kaiyan Zhang, Qionglei Zhong, Siying Chen, Chuanxian Guo, Yan Xu, Yang Liu, Wen Sun, Yijie Yan, Puning Zhao

**Affiliations:** Department of Ophthalmology, Hainan Provincial People’s Hospital, Haikou, 570311 China

**Keywords:** Age-related macular degeneration, Aged population, Hainan Province, Risk factor

## Abstract

**Purpose:**

The aim of this study was to investigate the prevalence of age-related macular degeneration (AMD) and the risk factors in the residents aged ≥50 years in Hainan Province.

**Methods:**

Random sampling was carried out in four separated cities in Hainan Province in 2015. All the subjects accomplished the standard questionnaire and ocular examinations. The diagnosis of AMD was performed based on the criteria proposed by Beckman Initiative for Macular Research Classification Committee.

**Results:**

Three hundred and fifty-seven subjects (15.6%) were diagnosed with AMD, including 267 (11.7%) of early AMD, 64 (2.80%) of intermediate AMD and 24 (1.1%) of late AMD, respectively. The factors associated with the prevalence of AMD included age, educational level, smoking, outdoor activities and diet. The prevalence of AMD increased with age, lower educational level, smoking or less outdoor activities. The prevalence of AMD in those with a diet of meat or eggs was higher compared with a diet of vegetables or fish. The prevalence of early, intermediate and late AMD in the aged population in Hainan Province was 11.7, 2.8 and 1.1%, respectively.

**Conclusions:**

Age and smoking were the risk factors for AMD, while the educational level and outdoor activities were the protective factors. Early AMD mostly occurred in those aged 50–59 years and 60–69 years, while intermediate and late AMD occurred in 70–79 years and older than 80 years.

## Introduction

Age-related macular degeneration (AMD) is one of the ocular diseases resulting in a higher vision loss rate or even blindness worldwide [1]. Nowadays, the prevalence of AMD shows gradual increase in China with the aging process. However, population based data on the prevalence and causes of AMD are limited throughout the world.

Although several factors have been reported to be responsible for the pathogenesis of AMD, such as smoking, sunshine, drinking alcohol, hypertension, high body mass index, diabetes, age and race [2], the pathogenesis of AMD is still not well defined. To characterize the potential causes and risk factors of visual loss or impairment, extensive emphasis has been paid on the rural population since 2006 as a majority of population are living in rural areas in China. Hainan province in southern China is localized in the tropical area with a long sunshine exposure. The agriculture is the major industrial structure, and the resident constitution is unique compared with the northern provinces in China as it is consisted of Han Chinese, Miao minority and Li minority. In China, a few studies have been carried out to investigate the prevalence of AMD, such as the Beijing eye study [3], the Handan eye study [4] and the Shipai eye study [5]. However, all these studies were mainly focused on the participants in northern China. In this study, we aim to investigate the epidemiological features of AMD in Hainan Province. Our study focused on the epidemiological features of AMD and the selection of risk factors.

## Materials and methods

### Subjects

The sample size was based on estimating an anticipated prevalence rate of 15.5% in AMD, according to Shanghai Jing’an District within an error bound of 2% for 95% confidence interval (CI) [6]. A sample of 2094 subjects aged ≥50 years were required for the sampling, assuming an examination response rate of 90% and a design effect of 1.5 accounting for inefficiencies associated with the cluster sampling design. The study protocols were approved by the Ethical Committee of Hainan Provincial People’s Hospital.

In this study, four separated cities (i.e., Haikou City, Sanya City, Wanning City and Dongfang City) localized in the North, South, East and West of Hainan province were designated as the sampling sites. These sites could represent the economic levels and include individuals of Han Chinese and minorities (e.g., Li minority and Miao minority). Four communities/villages were randomly selected from each district/town randomly selected from each city. In all these four communities/villages, subjects met the following aspects were eligible to this study: those aged ≥50 years; long-term residence or individuals living in the community/village at least 12 months. A computer-based random number generator was used for the sampling in each site, and 600 subjects were randomly selected. All the 2400 subjects were informed to participated in the ocular examination at the designated sites. For those absent from the examination, a door-to-door visit would be given. In total, 2288 subjects participated in this study, among which 56 subjects were excluded due to blurred fundus in any eye. Finally, 2232 subjects were included in this study.

## Methods

All the included subjects were required to fill in the questionnaire listed in Table 1. Besides, ocular examinations were performed for each subject, including: (1) visual acuity (VA) and best corrected visual acuity (BCVA); (2) anterior segment examination including cornea, pupils and lens by slit lamp microscope; (3) fundus examination using a slit lamp front mirror after mydriasis by compound tropicamide eye drops (tropicamide, 5 mg, plus phenylephrine, 5 mg/1 ml). Subjects with a history of glaucoma and/or a shallow anterior chamber were examined nonmydriatically. Those with fundus not clearly observed were excluded. The diagnostic criteria of AMD were based on the Beckman Initiative for Macular Research Classification Committee [7]. Early AMD was defined as drusen (65–125 μm) with no pigment abnormal. Intermediate AMD was defined as presence of at least one drusen (≥125 μm) with any retinal pigment epithelium (RPE) abnormalities. Late AMD was defined as formation of neovessels and geographic atrophy. The results of fundus examination were evaluated by two experienced retina ophthalmologists. In cases of disputes, a detailed discussion was held until reaching a consensus.

**Table 1:** Univariate analysis of the prevalence of AMD

Variables	Group	AMD	Non-AMD	Statistics	*P* value
		59	635	*χ* ^2^ = 43.546	<0.001
	60–69	142	555		
	70–79	105	480		
	≥80	51	205		
Sex	M	142	625	*χ* ^2^ = 5.519	0.019
	F	215	1250		
Ethnicity	Han ethnic group	308	1558	*χ* ^2^ = 20.527	<0.001
	Li ethnic group	35	294		
	Miao ethnic group	14	23		
Education	Junior school or less	245	991	*χ* ^2^ = 30.380	<0.001
	Middle school	74	563		
	High school or more	38	321		
Hypertension	Y	148	682	*χ* ^2^ = 3.318	0.069
	N	209	1193		
BMI	<18.5	65	339	*χ* ^2^ = 0.984	0.611
	[18.5–24)	222	1125		
	≥24	70	411		
Diet	Meat	78	256	*χ* ^2^ = 26.478	<0.001
	Egg	10	18		
	Fish	38	186		
	Vegetables	79	495		
	Mixed	152	920		
Drinking tea	Never, or less than once per week	152	760	*χ* ^2^ = 0.518	0.472
	Y	205	1115		
Drinking alcohol	Never, or less than 50 g per week	294	1628	*χ* ^2^ = 7.248	0.064
	≤50 g per day	36	118		
	100–250 g per day	25	117		
	≥250 g per day	2	12		
Smoking	Never, or less than 1 cigarette per week	294	1750	*χ* ^2^ = 80.886	<0.001
	<20 cigarettes per day	31	85		
	20–40 cigarettes per day	23	40		
	>60 cigarettes per day	9	0		
Outdoor exercise	None	105	415	*χ* ^2^ = 12.203	0.007
	<2 h per day	81	406		
	2–5 h per day	98	658		
	>5 h per day	73	396		
Systemic disease	None	249	1289	*χ* ^2^ = 3.577	0.466
	Cardiovascular and cerebrovascular diseases	52	228		
	Hyperlipemia	11	67		
	Diabetes	6	48		
	Other disease	39	243		
Surgical history	None	309	1650	*χ* ^2^ = 2.032	0.566
	Intraocular surgery	35	150		
	Extraocular surgery	11	69		
	Others	2	6		

The prevalence of AMD was calculated as the percentage of subjects with at least one eye confirmed with AMD. In cases of a patient confirmed with AMD in both eyes, the more severe one was used to represent the staging of the disease.

### Statistical analysis

SPSS17.0 software was used for the data analysis. Chi-square test or rank test was performed for the comparison of numeration data. Logistic regression analysis was performed to identify the risk factors of AMD. *P* < 0.05 was considered to be statistical significance.

## Results

### Subjects characteristics

In total, 2288 subjects were included in this study. Fifty-six were excluded due to fundus blur in any eye (e.g., cataract, corneal disease and vitreous opacity). Finally, 2232 subjects (97.6%) (male: 767, female: 1405; mean age: 65.79 years) accomplished the test. Three hundred and fifty-seven subjects (15.6%) were diagnosed with AMD, including 267 (11.7%) of early AMD, 64 (2.80%) of intermediate AMD and 24 (1.1%) of late AMD, respectively.

### Factors associated with the prevalence of AMD in Hainan Province

A remarkable difference was noticed in the prevalence of AMD among the subjects aged 50–59, 60–69, 70–79 years and older than 80 years, respectively (*χ* ^2^ = 43.546, *P* < 0.001, Table 1). Furthermore, linear trend test showed that the prevalence of AMD increased with age (*χ* ^2^ = 23.280, *P* < 0.001). After adjusting Bonferroni–corrected alpha level (0.05/3 = 0.0167), the rank sum test was used to analyze the difference of age distribution between early, intermediate and late AMD patients, respectively. The results showed that early AMD mostly occurred in 50–59 years and 60–69 years compared with intermediate (*Z* = −3.625, *P* < 0.001), or late AMD (*Z* = −4.619, *P* < 0.001) mostly occurred in 70–79 years and older than 80 years, respectively (Table 2). No statistical difference was noticed in the prevalence of intermediate and late AMD in those aged 70–79 years and older than 80 years (*Z* = −1.824, *P* = 0.068). The prevalence of AMD in the male subjects was higher than that of the female counterparts (18.5 vs. 14.7%, *P* = 0.019). The Bonferroni method was used to correct the test level of alpha (0.05/3 = 0.0167), and the difference of the prevalence in AMD between ethnicity was analyzed. The prevalence of AMD was higher in the Miao ethnic group than that of the Han ethnic group (*χ* ^2^ = 11.744, *P* = 0.001), and the Li ethnic group (*χ* ^2^ = 21.220, *P* < 0.001). The prevalence of AMD was higher in the Han ethnic group compared with that of Li ethnic group (*χ* ^2^ = 7.303, *P* = 0.007). Significant differences were noticed in the prevalence of AMD in the subjects with different educational levels. Linear trend test showed that the prevalence of AMD was decreased in subjects with higher education level (*χ* ^2^ = 26.490, *P* < 0.001). Subjects living on a diet of meat or egg showed a higher prevalence of AMD compared with those on a diet of fish or vegetables (*α* = 0.05/10 = 0.005, *P* < 0.0045). The linear trend test showed that the prevalence of AMD increased with smoking (*χ* ^2^ = 66.393, *P* < 0.001), while decreased with longer time for outdoor activities (*χ* ^2^ = 6.983, *P* = 0.008).

**Table 2:** Age of the subjects with early, intermediate and late AMD

Stage	50–59 years	60–69 years	70–79 years	≥80 years	Statistics	*P* value
Early AMD	49	124	67	27	*H* = 30.167	<0.001
Intermediate AMD	10	12	28	14		
Late AMD	0	6	10	10		

According to the univariate analysis by Chi-square test and rank test, no association was identified between the prevalence of AMD and hypertension, body mass index, drinking tea or alcohol, diabetes or other systemic disease, or a history of surgery (*P* > 0.05, Table 1).

### Logistic regression analysis

Logistic regression analysis was performed based on the results obtained in the univariate analysis, using AMD as the dependent variable (no AMD = 0, AMD = 1). The independent variables included age, educational level, drinking alcohol, smoking, outdoor activity, blood pressure, sex, ethnicity (Li ethnic group = 1, Miao ethnic group = 2, Han ethnic group = 3) (dummy variable, Han Chinese is the reference level), and diet (a diet of meat = 1, a diet of egg = 2, a diet of fish = 3, a diet of vegetables = 4, a mixed diet = 5) (dummy variable, Mixed diet is the reference level). Finally, six factors included in the regression analysis, including age, educational level, smoking, outdoor activity, ethnicity and diet (*P* < 0.05, Table 3).

**Table 3:** Logistic regression analysis of AMD subjects aged >50 years in Hainan Province

Variable	*B*	Standard error	Wald	*P*	OR	95.0% CI for EXP (*B*)	
Age	0.292	0.061	22.524	0.000	1.339	1.187	1.510
Education	−0.319	0.088	13.078	0.000	0.727	0.611	0.864
Drinking alcohol	0.110	0.110	1.591	0.207	0.871	0.702	1.080
Smoking	0.762	0.123	38.426	0.000	2.143	1.684	2.726
Outdoor exercise	−0.154	0.054	7.994	0.005	0.858	0.771	0.954
Sex	−0.115	0.133	0.754	0.385	0.891	0.687	1.156
Blood pressure	0.212	0.123	2.983	0.084	1.237	0.972	1.573
Ethnicity	-	-	10.815	0.004	-	-	-
Li ethnic group	−1.150	0.394	8.527	0.003	0.317	0.146	0.685
Miao ethnic group	−1.335	0.406	10.810	0.001	0.263	0.119	0.583
Diet	-	-	8.372	0.045	-	-	-
A diet of meat	0.321	0.162	3.916	0.042	1.379	1.003	1.896
A diet of egg	0.898	0.417	4.630	0.031	2.454	1.083	5.559
A diet of fish	0.131	0.202	0.419	0.518	1.140	0.767	1.694
A diet of vegetables	−0.008	0.147	0.003	0.959	0.992	0.744	1.324
Constant	−1.058	0.634	2.783	0.095	0.347	-	-

After the logistic regression model was built, multivariate ordered logistic regression analysis was carried out to evaluate the prevalence of AMD using age, education level, alcohol drinking, smoking, outdoor exercise, sex, ethnicity, blood pressure and diet as the independent variables. The results showed that the following independent variables were entered into the equation (*P* ≤ 0.05), including age, education level, smoking, outdoor activities, ethnicity and diet.

Logistic regression analysis indicated several factors were correlated with the prevalence of AMD, including age, education level, smoking, outdoor activities, ethnicity and diet (*P* < 0.05, Table 3). Among these factors, prevalence of AMD was positively correlated to the age (*B* = 0.212) and smoking (*B* = 0.873), respectively. Meanwhile, educational level (*B* = −0.369) and outdoor activities (*B* = −0.132) were negatively correlated to the prevalence of AMD. On the contrary, sex, blood pressure and drinking alcohol were not the risk factors of AMD (*P* > 0.05). The risk factors of AMD were age (OR 1.236, 95.0% CI 1.091, 1.401) and smoking (OR 2.394, 95.0% CI 1.925, 2.977). Higher education level was a protective factor for the AMD (OR 0.691, 95.0% CI 0.575, 0.830), together with outdoor activities (OR 1.14, 95.0% CI 1.005, 1.297). The subjects of Li minority showed a lower prevalence of AMD (OR 0.581, 95.0% CI 0.386, 0.875) compared to the Han Chinese, but the subjects of Miao minority showed a higher prevalence of AMD. A diet of meat or eggs was the risk factor for AMD (meat: OR 1.811, 95.0% CI 1.322, 2.482; egg: OR 2.492, 95.0% CI 1.095, 5.663, Table 2). In contrast, a diet of vegetables or fish was not the risk factor for AMD (*P* > 0.05).

## Discussion

No study has been carried out to investigate the epidemiological features of AMD in Hainan Province. In this study, we firstly reported the epidemiological features of AMD in the province and revealed the potential risk factors and protective factors of AMD.

Hainan province (with a latitude of 18°–20°) was surrounded by sea at four sides with a long sunshine duration (more than 2000 h annually) and a high quantity of radiant energy (110,000–120,000 cal). The agriculture is the major industrial structure of the province. Most of the residents were farmers with a lower education. On this basis, individuals aged more than 20 years were considered to be the major labor force for the family, and many of them had to work until aged. Meanwhile, the medical level is comparatively lower, which hampers the management of ocular diseases.

In this study, the prevalence of AMD was 15.6% among the included subjects, among which 267 eyes (11.7%) were confirmed with early AMD, 64 eyes (2.80%) with intermediate AMD, and 24 eyes (1.1%) with late AMD, respectively. Up to now, several studies have been carried out on the survey of AMD [8, 9], but there are still disputes on the prevalence of AMD in the population. For example, the prevalence of AMD in the population aged ≥50 years in Japan was 8.5% for the early AMD and 0.8 for the late AMD [8]. However, the percentages in the Republic of Korea [9], Ireland [10] and Kenya [11] were 5.07% and 0.34, 6.6 and 0.6%, as well as 11.2 and 1.2%, respectively, whereas for the prevalence of AMD in the population aged ≥40 years in China [3], Singapore [9] and the USA [12], the percentages were 1.4% and 0.2, 3.5 and 0.34%, as well as a total rate of 1.47%, respectively.

Age was considered as the most important risk factor for AMD. Mukesh et al. reported the prevalence of AMD was 0.20% in the subjects aged 55–64 years, while it was sharply elevated to 13.00% among the population aged more than 85 years [13]. Besides, the prevalence of exudative AMD was elevated from 0.71 to 5.80%, together with a prevalence of geographic atrophy from 0.04 to 4.20%. In our study, the incidence of AMD was positively related to the age of the subjects. Exactly, a remarkable increase was noticed the prevalence of early, intermediate and late AMD in these subjects. Besides, subjects aged 50–59 years and 60–69 years showed a higher prevalence of early AMD, while those aged 70–79 years and ≥80 years were more likely to develop intermediate and late AMD.

The female are apt to develop AMD compared with the male counterparts [14, 15]. In our study, univariate analysis indicated the prevalence of AMD was higher in the male population compared to the female counterpart, whereas, in the multivariate analysis, no correlation was noticed between sex and the prevalence of AMD. This may be related to the fact that more female participated in the survey.

In this study, we investigated the prevalence of AMD in the minorities in the Hainan Province. The prevalence of AMD in the Miao ethnic group was higher than that of the Li ethnic group and Han ethnic group. Meanwhile, the prevalence of AMD in the Han ethnic group was higher than that of the Li ethnic group. For the reason, most of the Miao ethnic group individuals have a favor of eating dog meat and souse food. Besides, the educational levels were comparatively lower compared with the other populations. However, further studies are needed to validate whether AMD is correlated to these factors.

In line with the previous studies [16, 17], our results confirmed lower education was also a risk factor for the AMD. To explain this, we speculated that most of the individuals with lower education worked in conditions that were labor-intensive (such as farm work) and long-term high intensity sunshine exposure. Besides, these individuals are reported to show a higher dietary intake of fat, while the intake of substances that are required to keep fit such as folacin, magnesium and vitamin C is comparatively lower [18]. All these factors may lead to the formation of atheromatous plaque and stenosis in the blood vessels, which results in inefficient blood supply in the choroid and subsequent AMD due to dysfunction of RPE. Interestingly, individuals of lower education usually smoke, which is also a reason for the higher prevalence of AMD [19].

Dietary intake of lutein or zeaxanthin [20], carotene [21, 22], low sugar and grains [23] or fish [24, 25] has been reported to be associated with decrease in AMD. Among these components, lutein, zeaxanthin or carotene showed anti-oxidant capacity, which may effectively attenuate the oxidative damages of the retina. Some unsaturated fatty acids (i.e., DHA and EPA) abundant in the fish meat have been approved to play important roles in regulating the inflammation and immunological reaction, and these components may affect the pathogenesis of AMD in vivo [26]. On the contrary, individuals with frequent intake of red meat and trans unsaturated fatty acid [27, 28] showed a higher prevalence of AMD, which may be related to the accumulation of endogenous nitroso compounds [29, 30] that may cause toxicities to the retina due to degrading into the methylated substances [31]. In this study, we found a diet of eggs or meat was the risk factor for AMD, while those on a diet of fish and/or vegetables showed remarkably decrease in the prevalence of AMD. Caffeine, the major content in tea, was reported to affect the function of multiple organs, such as increasing the excitability of the central nervous system and heart rate, as well as contributing to the cardiac systole. To date, there is no consensus on the correlation between drinking tea and the prevalence of AMD [32, 33]. Our results showed drinking tea was not related to the prevalence of AMD.

To date, there are still disputes on the roles of drinking alcohol in the prevalence of AMD. Some studies proposed that alcohol was related to the macular degeneration in Latinos [34, 35]; however, in a prospective analysis, Boekhoorn et al. reported that alcohol was not a risk factor for AMD [36]. In this study, alcohol was not related to the prevalence of AMD, which was presented as decrease in thrombosis in the choroid in the individuals drinking alcohol than those drinking no alcohol [37].

Nowadays, smoking has been regarded as a risk factor for AMD. According to a recent survey in China in 2015, the percentages of smoking individuals aged 15–24, 25–44, 45–64 years and 65–69 years were 13.5, 20.9, 26.1 and 27.2%, respectively. Smoking was considered to be responsible for the formation of choroidal neovascularization [38]. Besides, it may involve in inducing AMD through elevating the oxidative stress, reducing the extent of anti-oxidant, or alternating the vascular structures [39]. Our results showed individuals with smoking showed a higher prevalence of AMD than those with no smoking. Besides, in line with the previous studies [39–41], our study showed the prevalence of AMD increased with the elevation of pack of cigarettes per day.

Logistic regression analysis revealed outdoor activities were negatively correlated to the prevalence of AMD, which was not in line with the previous description that extended sunshine contributing to a higher prevalence of AMD [38]. For the reason, sunshine may cause injury to the pigment epithelium layer, which consequently resulted in AMD [41]. However, exposure to ultraviolet may increase the prevalence of cataract, which may hamper the penetration of sunshine into the fundus. This may decrease the prevalence of AMD.

Previous studies showed hypertension and higher BMI was the risk factor for the AMD [42, 43]; however, our results showed these factors were not associated with the pathogenesis of AMD. In addition, systemic conditions, such as cerebrovascular diseases, diabetes mellitus [44–46], were considered as the risk factors for AMD, which was associated with the vascular lesions that may result in AMD. Nevertheless, our results revealed these factors were not the risk factor for AMD in the residents in Hainan Province. In a previous study, Pham et al. [47] revealed pterygium was associated with an increased risk of early and late AMD, which may be related to the excessive vascular growth factors that finally induced exudative AMD. In this study, individuals with histories of ocular surgery showed no difference in the prevalence of AMD compared with those without. In future, large sample size studies are needed to validate such correlation.

Indeed, there are limitations in our study. Bias may present to the analysis due to memory loss in the subjects aged >50 years when describing their personal histories. Besides, most of the subjects were of lower education, with poor awareness of the AMD. This may induce bias for the survey.

In conclusion, the prevalence of early, intermediate and late AMD in the aged population in Hainan Province was 11.7, 2.8 and 1.1%, respectively. The prevalence was higher than the other populations in the Asian countries. High educational level and outdoor activities were protective factors for AMD, while the pathogenesis of AMD was found to be correlated with age, smoking and a diet of meat and eggs.
